# Effects of 0.15% ropivacaine alone and combination with sufentanil on epidural labor analgesia and adverse reactions

**DOI:** 10.4314/ahs.v23i3.66

**Published:** 2023-09

**Authors:** Huanhui Zhong, Yongdong Wang, Yiqun Wang, Heng Li

**Affiliations:** Department of Anesthesiology, Affiliated Nanhua Hospital, University of South China, Hengyang 421002, Hunan Province, China

**Keywords:** Ropivacaine, sufentanil, epidural labor analgesia, adverse reaction

## Abstract

**Objective:**

The aim of this study was to compare the impacts of 0.15% ropivacaine alone and 0.15% ropivacaine combined with sufentanil on epidural labor analgesia.

**Methods:**

A total of 297 eligible primiparae were randomly divided into group A (n=149, 0.15% ropivacaine + sufentanil) and group B (n=148, 0.15% ropivacaine). Visual analogue scale (VAS) scores prior to analgesia and 20 min following epidural medication, the maximum VAS score during labor, dosage of analgesic drugs, modified Bromage score, satisfaction degree, labor duration, delivery mode, 1-min and 5-min Apgar scores of newborns, adverse reactions during analgesia, and fever during labor were recorded.

**Results:**

Group A and B had similar VAS scores 20 min following epidural medication and maximum score during labor (P>0.05), which significantly fell compared with those before labor analgesia (P<0.05). The occurrence rates of nausea and vomiting were of significant difference (P<0.05).

**Conclusion:**

0.15% ropivacaine alone achieves a comparable epidural labor analgesia effect to that of 0.15% ropivacaine + 0.05 µg/mL sufentanil on primiparae.

## Introduction

Delivery is a natural physiological process of females. In the early stage of labor, regular uterine contraction and cervical dilatation induce pain, which can trigger hemodynamic changes such as elevated heart rate and blood pressure, posing obvious physiological and psychological burdens on parturient. Labor analgesia helps improve parturient' feeling during delivery and the pregnancy outcome, thus reducing the cesarean section rate caused by intolerance to pain^1^.

Epidural block is recognized as the first choice for labor analgesia worldwide^2^. Currently, the epidural drugs recommended in the guidelines of the American College of Obstetricians and Gynecologists are local anesthetics alone or in combination with opioids.^3^ However, the need for compound opioids and the concentration of local anesthetics are still controversial. Compared with ropivacaine alone, epidural ropivacaine combined with sufentanil has better analgesic effect and smaller local anesthetic dose^4^, but aggravates the adverse reactions of parturients undergoing epidural labor analgesia. Opioids can prolong labor, cause urinary retention and pruritus^5^, and may also reduce the Apgar score of newborn^6^. At present, there is no evidence-based medicine basis for the adverse reactions of local anesthetics alone and in combination with opioids during labor analgesia.

In this study, therefore, the clinical epidural labor analgesia effect and adverse reactions in primiparae were compared between ropivacaine alone and in combination with sufentanil, aiming to provide references for the selection of anesthetic drugs for epidural labor analgesia.

## Materials and methods

### Sample size estimation

The sample size was calculated based on the maximum VAS score during labor. According to the study of Zhou *et al^7^*, when 0.15% ropivacaine + 0.5 µg/mL sufentanil was applied for analgesia, the maximum VAS score during labor was (4.2±1.1) points. In the study of Wang *et al.*^4^, the VAS score of ropivacaine alone was 10% higher than that of ropivacaine combined with sufentanil. Assuming α=0.05 and β=0.2, 110 parturients were needed in each group. Considering withdrawal and data loss, it was planned to enrol 150 parturients for each group, with a total of 300 parturients.

### General information

This study has been approved by the ethic committee of our hospital, and written informed consent has been obtained from all patients or their family members. Full-term primiparae hospitalized for natural delivery from November 1, 2018 to August 31, 2019 were enrolled. These primiparae, aged 22-41 years old, with a gestational age of 31-42 weeks and ASA grade I or II, were admitted to our hospital for natural delivery (singleton, cephalic position, labor analgesia planned). Patients with preeclampsia, BMI >35 kg/m^2^, contraindication of intraspinal anesthesia, or allergy to opioids or ropivacaine, or those who refused to participate in this study were excluded. The exclusion criteria were as follows: (1) patients whose level of anesthesia failed to reach T10 at 30 min after epidural medication, (2) those with accidental dural puncture, general spinal anesthesia or local anesthetic poisoning during epidural puncture, (3) those with analgesia pump failure during labor analgesia, or (4) those with epidural catheter blockage or accidental prolapse during labor analgesia.

The parturients were divided into groups A and B using the random number table method. The experimental drugs were specially prepared by a researcher who was not involved in the subsequent anesthetic procedures and follow-up. Patients in groups A and B were given 0.15% ropivacaine (Sigma-Aldrich, USA) combined with 0.5 µg/ mL sufentanil (Sigma-Aldrich, USA) and 0.15% ropivacaine alone, respectively. This is a single-blind study, in which all operations and follow-up were carried out by anesthesiologists who did not know about the grouping.

### Analgesia methods

Once the parturients entered the labor stage, their peripheral vein was opened. Then analgesia was conducted when the uterine mouth size was ≥2 cm. After successful epidural puncture through L_2-3_ space, 4-5 cm of tube was placed at the head end, and 3 mL of 1.13% lidocaine carbonate (Sigma-Aldrich, USA) was administered. If there was no general spinal anesthesia or local anesthetic poisoning at 5 min later, 10 mL of epidural analgesic drug was given as the induction dose. At 15 min after administration of induction dose, the level of anesthesia was measured using an alcohol cotton ball. After the level of anesthesia exceeded T_10_, the patient-controlled analgesia (PCA) pump (Jiangsu Aipeng Medical Technology Co., Ltd., China) was connected. The background dose was 8 mL/h, the PCA dose was 8 mL, and the locking time was 15 min. If there was breakthrough pain during labor [visual analogue scale (VAS) score >4 points], the anesthesiologist examined whether the epidural catheter was in the correct position, and then gave 5 mL of 0.2% ropivacaine for remedial analgesia. If the pain was not relieved after 15 min, the analgesia was considered failed. The anesthesiologist examined the level of anesthesia, identified the causes, and re-punctured or adjusted the formula. Finally, the case was withdrawn from this study.

### Observation indices

The VAS scores before analgesia and at 20 min after epidural medication, and the maximum VAS score during labor were recorded. Besides, the data of labor analgesia, including the times of PCA pump pressing and remedial analgesia, dosage of analgesics, modified Bromage score after induction (0 point = no motor block, and full flexion of hip, knee and ankle joints, 1 point = unable to flex hip joint, could not complete straight leg lift, but able to flex knee and ankle joints fully, 2 points = unable to flex knee joint, only able to flex ankle joint, 3 points = unable to flex ankle joint, or unable to move the lower limbs), withdrawal due to analgesia failure, and analgesia satisfaction rate, were recorded. Moreover, the duration of labor, mode of delivery, 1-min and 5-min Apgar scores of newborns, number of cases with neonatal Apgar score ≤7 points, and incidence rates of pruritus, nausea and vomiting, urinary retention, fever during labor (body temperature ≥37.5^0^C at any time during labor) and sleepiness were recorded.

### Statistical analysis

SPSS26.0 software (IBM Inc., USA) was utilized for statistical analysis. The measurement data in normal distribution were expressed as mean ± standard deviation, and one-way ANOVA was performed for the comparison between groups. The measurement data in non-normal distribution were expressed as median (M) and interquartile range (IQR), and the Mann-Whitney U test was conducted for the comparison between groups. The count data were expressed as [n (%)], and the χ^2^ test or Fisher exact test was performed for the comparison between groups. P<0.05 suggested that the difference was statistically significant.

## Results

### Baseline clinical data

There were 308 parturients meeting the inclusion criteria, of whom 6 were excluded (4 cases with preeclampsia and the other 2 cases with BMI >35 kg/m^2^). Finally, a total of 300 parturients were enrolled (n=150). During the study, 1 parturient in group B was withdrawn from the study due to analgesic pump failure, and 1 parturient in each group was withdrawn from the study because of accidental puncture of the spinal dura mater. There were no significant differences in age, height, weight, gestational age and uterine mouth size during analgesia between the two groups (P>0.05) (Table 1).

**Table 1:** Baseline clinical data

Item	Group A	Group B	*t*	P
	(n=149)	(n=148)		
Age (Y)	28.78±4.34	28.83±4.41	0.098	0.922
Height (cm)	162.03±4.67	161.18±4.58	1.584	0.114
Weight (kg)	71.27±6.47	71.34±6.53	0.093	0.926
Gestational age (week)	39.8±1.0	39.7±1.0	0.862	0.390
Uterine mouth size (cm)	2.27±0.45	2.23±0.51	0.717	0.474

### VAS scores at different time points

Before labor analgesia, there was no significant difference in the VAS score between the two groups (P>0.05). At 20 min after epidural medication, the VAS score was significantly lower than that before analgesia in both groups (P<0.05). The maximum VAS score during labor was significantly lower than that before analgesia in both groups (P<0.05), but there was no significant difference between the two groups (P>0.05) (Table 2).

**Table 2:** VAS scores at different time points [point, M (IQR)]

Group	Before analgesia	20 min after epidural medication	Maximum during labor
Group A (n=149)	8 (8~9)	1 (1~2) *	2 (2~3) *
Group B (n=148)	8 (7~9)	1 (1~2) *	2 (2~3) *

* P<0.05 *vs.* before analgesia.

### Labor analgesia-related data

No significant differences were observed in the times of PCA pump pressing, dosage of analgesics and modified Bromage score between the two groups (P>0.05). Besides, there was no significant difference in the number of cases requiring remedial analgesia between the two groups (P>0.05). In the study period, there was no analgesia failure in both groups. No significant difference was found in satisfaction degree toward analgesia between the two groups (P>0.05) (Table 3).

**Table 3:** Labor analgesia-related data

Index	Group A (n=149)	Group B (n=148)
Times of PCA pump pressing (n)	3 (2~5)	3 (2~5)
Dosage of analgesics (mL)	18.40 (51.0~99.0)	83.10 (56.0~104.0)
Dosage of ropivacaine (mg)	18.40 (51.0~99.0)	124.10 (84.0~156.0) *
Modified Bromage score [n (%)]		
0 point	141 (98.66)	146 (98.65)
1 point	2 (1.34)	2 (1.35)
Remedial analgesia [n (%)]		
0	134 (89.93)	136 (91.89)
Once	6 (4.03)	2 (1.35)
Twice	9 (6.04)	9 (6.08)
3 times	0 (0.00)	1 (0.68)
Satisfaction degree toward analgesia (%)	142 (95.30)	142 (95.95)

* P<0.05 *vs.* group A.

### Delivery outcomes

No significant differences were observed in the durations of the first and second stages of labor and the mode of delivery between the two groups (P>0.05). The Apgar scores of newborns were above 9 points in both groups, displaying no significant difference between the two groups (P>0.05) (Table 4).

**Table 4:** Delivery outcomes

Index	Group A (n=149)	Group B (n=148)
Duration of the first stage of labor (h)	10.0 (1.0~12.5)	10.0 (6.5~13.5)
Duration of the second stage of labor (h)	1.2 (0.6~1.1)	1.3 (0.1~1.8)
Mode of delivery [n (%)]		
Natural labor	106 (11.14)	108 (12.91)
Forceps delivery	18 (12.08)	15 (10.14)
Cesarean section	25 (16.18)	25 (16.89)

### Incidence of adverse reactions

There were 13 cases (8.12%) of pruritus in group A, including 10 cases of mild itching and 3 cases of severe itching, who were relieved after intravenous injection of naloxone (0.1 mg), while no pruritus was reported in group B (P<0.05). There were no significant differences in the incidence rates of nausea and vomiting, urinary retention and fever during labor between the two groups (P>0.05). Besides, no sleepiness was observed in both groups (Table 5).

**Table 5:** Incidence of adverse reactions [n (%)]

Index	Group A (n=149)	Group B (n=148)
Pruritus	13 (8.72)	0 (0.00) *
Nausea and vomiting	7 (4.70)	5 (3.38)
Urinary retention	12 (8.05)	6 (4.05)
Fever during labor	32 (21.48)	33 (22.30)

* P<0.05 *vs.* group A.

## Discussion

Compared with other methods of analgesia, epidural labor analgesia is able to relieve pain during labor and improve maternal satisfaction more effectively^8^. Therefore, it is a commonly used method of labor analgesia in China and abroad. The typically used epidural analgesics include local anesthetics and local anesthetics combined with opioids^3^. Epidural application of opioids can exert a synergistic effect with local anesthetics, thus reducing the occurrence of motor block, decreasing the dosage of local anesthetics, and extending the duration of analgesics by lowering the concentration of local anesthetics. However, opioids will lead to some adverse reactions, such as pruritus and urinary retention in the parturients^9^, and a decreased Apgar score, respiratory depression and increased total adverse reactions in the newborns^4^.

Ropivacaine combined with sufentanil is extensively used in labor analgesia, and its effectiveness has been confirmed widely. 0.15% ropivacaine + 0.5 µg/mL sufentanil has definite analgesic effect and few adverse reactions^10^, and it is a routine epidural labor analgesia formula in our hospital, so this formula was adopted in group A (control group). Gouez et al. found that the lowest effective concentration of ropivacaine for labor analgesia in Chinese population was 0.154%^11^, so 0.15% ropivacaine was applied in group B (study group). The results of this study manifested that the analgesic effect was good and comparable in the first stage of labor in both groups, and there were no obvious differences in the VAS score and number of PCA between the two groups. In the later period of the first stage of labor, the intensity of uterine contraction increases, the interval of uterine contraction shortens, and the pain is much more severe than at the beginning of the labor stage. Even if epidural labor analgesia has been performed, there will often be breakthrough pain. The occurrence of breakthrough pain not only seriously affects maternal satisfaction, but also requires additional remedies to deal with it, increasing the workload of anesthesiologists. In this study, good analgesic effects have been achieved in the later stage of labor in both groups, so there was less need for remedial analgesia. In the study of Sng *et al.*^12^, 0.2% ropivacaine was able to bring good analgesic effects and no adverse reactions, in line with the results of this study. In the present study, the remedial effect of 0.2% ropivacaine was better, most of the parturients only needed one remedy, and none of them was withdrawn from the study due to remedy failure. However, there were still some parturients who needed two or more remedies. After remedial analgesia, no change in motor block was observed in both groups.

Ropivacaine has low cardiotoxicity and is featured with sensorimotor block separation at low concentration. When its concentration is less than 0.17%, the motor block is mild. Since 0.15% ropivacaine triggers mild motor block, only 2 parturients had modified Bromage score >0 point. Wang *et al.* found in a large single-center, double-blind, randomized, controlled study that the 1-min Apgar score of newborns was lower, and the number of newborns with an Apgar score ≤7 points were greater in sufentanil + ropivacaine group (parturients underwent epidural labor analgesia with 0.125% ropivacaine + 0.3 µg/mL sufentanil) than those in simple ropivacaine group (parturients underwent epidural labor analgesia with 0.125% ropivacaine alone)^4^, which is in contradiction with the results of this study. In the present study, there was no significant difference in the Apgar score between the two groups, and Apgar score ≤7 points was not observed in both groups. This may be related to the different obstetrical procedures in the hospital. Our hospital may have more active management of neonatal intrauterine distress, which can be reflected by the higher rates of cesarean section and instrumental delivery.

In this study, 13 parturients in group A had pruritus, and it was so severe in 3 of them that drug intervention was needed, while no pruritus was reported in group B. This is consistent with the study of Bernard *et al.* that epidural sufentanil dramatically elevated the incidence rate of maternal pruritus which also increased with rising sufentanil concentration^5^. At present, pruritus caused by the application of opioids in the spinal canal may mainly be attributed to the following mechanisms. (1) Activation of µ-receptor: Opioid drugs activate spinal cord opioid µ-receptors, which not only play an analgesic role, but also activate central µ-receptors to trigger central pruritus^13^. (2) Activation of gastrin-releasing peptide receptor (GRPR) in cornu dorsale medullae spinalis: GRPR in cornu dorsale medullae spinalis + neurons can express GRPR, and specifically mediate itch transmission. The isomer formed by µ-receptor and GRPR is related to opioid-induced pruritus^14^. (3) Regulation of 5-hydroxytryptamine-3 (5-HT3) receptor: 5-HT3 can activate HTR7 receptor, which is closely related to pruritus, thus causingTR7 to open ion channel TRPA1 and resulting in pruritus^15^. (4) Activation of N-methyl-D-aspartate receptor (NMDAR): When NMDAR is activated by glutamate, it opens ion channels, triggering cell depolarization, activating a variety of intracellular signal molecules, and thus participating in the transmission of itch sensation in cornu dorsale medullae spinalis^16^. Although many studies have shown that intraspinal morphine causes pruritus, sufentanil may also cause pruritus through the above receptor pathways.

In summary, the epidural labor analgesia effect of simple 0.15% ropivacaine is comparable to that of 0.15% ropivacaine + 0.05 µg/mL sufentanil for primiparae, but its incidence of pruritus is decreased. Nevertheless, this study has some limitations. Firstly, no control of 0.15% ropivacaine + sufentanil was set. Boselli et al. reported that the application of 0.15% ropivacaine + 0.05 µg/mL sufentanil in labor analgesia showed no advantage compared with 0.1% ropivacaine + 0.05 µg/mL sufentanil, and the adverse reactions increased17, so no control was set in this study. Secondly, only short-term adverse reactions were observed, so more studies are needed for the analysis of long-term adverse reactions such as low back pain and postpartum depression. Thirdly, this was only a single-center study which only involved healthy singleton full-term parturients, so the conclusion needs to be further confirmed by multicenter studies with larger sample sizes.
